# Emerging Trimethoprim-Sulfamethoxazole Resistance Among Pathogens Isolated From Diabetic Foot Ulcers: A Case Emphasizing Early Culture-Guided Therapy

**DOI:** 10.7759/cureus.100346

**Published:** 2025-12-29

**Authors:** Jean Paul Ambroise, Azary Hernandez, Sarah DeHelian, Rafael A Gallardo, Rosa Huguet

**Affiliations:** 1 Medicine, Center for Haitian Studies, Miami, USA; 2 Internal Medicine, Jackson Memorial Hospital, Miami, USA; 3 Family Medicine, Center for Haitian Studies, Miami, USA; 4 Medicine, American University of the Caribbean, Miami, USA; 5 Family Medicine, St. George's University School of Medicine, True Blue, GRD

**Keywords:** antibiotic resistance, bacteroides fragilis, culture-guided therapy, delayed wound healing, diabetic foot ulcer, diabetic wound, mrsa infection, polymicrobial infection, trimethoprim-sulfamethoxazole

## Abstract

Lower extremity ulcers are a major complication of diabetic disease, often leading to a source of infection for patients. Due to delayed wound healing, ulcers quickly develop into extensive lesions leading to infections that are polymicrobial in nature and highly likely to acquire antibiotic resistance. We present the case of a 44-year-old man with poorly controlled type 2 diabetes mellitus who developed an ulcer on the left lower extremity after an episode of mild pruritus. Failure of early patient presentation to the office resulted in the propagation of infection, including areas of depth and necrosis. The ulcer was initially treated with trimethoprim-sulfamethoxazole (SXT) until cultures revealed a polymicrobial infection of *Bacteroides fragilis*, group B *Streptococcus*, and *Staphylococcus aureus*, with resistance to SXT observed exclusively in *S. aureus*. Furthermore, the ulcer continued to show signs of infection. As a result, medications were adjusted to metronidazole and ciprofloxacin, combined with meticulous wound care, lifestyle modifications, and optimization of glycemic and lipid control. After treatment modifications, the ulcer showed full-thickness healing. This case highlights the evolving challenge of antimicrobial resistance in skin and soft tissue infections. While SXT is commonly used for infected diabetic ulcers, the increasing prevalence of resistant *S. aureus* strains emphasizes the importance of culture-guided therapy, careful empiric antibiotic selection, and close patient follow-up. With this holistic, patient-centered approach in managing diabetic ulcers, physicians can catch antibiotic resistance early, preventing serious adverse outcomes and improving quality of life.

## Introduction

Diabetic ulcers are commonly observed in patients with long-standing, uncontrolled diabetes. Chronic hyperglycemia contributes to both microvascular and macrovascular complications, the former including retinopathy, neuropathy, and nephropathy [1]. Diabetic neuropathy is one of the two principal complications that predispose patients to ulcer formation [1]. Within one year of diagnosis, approximately 7% of patients with diabetes develop neuropathy, and the prevalence rises to nearly 50% after 25 years of disease duration [2].

Sustained hyperglycemia initiates a cascade of metabolic disturbances, including the activation of the polyol pathway, accumulation of advanced glycation end products, increased cytokine release, protein kinase C activation, and oxidative stress, all of which contribute to peripheral nerve injury [2]. This results in progressive nerve damage and impaired sensory perception, such as diminished ability to detect pain, heat, and cold. Peripheral nerves are particularly vulnerable to ischemia because their blood supply is limited; only a few transperineurial arterioles penetrate the endoneurium to nourish the nerve fibers [2]. Furthermore, these arterioles lack autoregulatory capacity, preventing compensatory increases in blood flow during periods of heightened metabolic demand [2].

Atherosclerosis is another one of the two principal complications that predispose patients to ulcer formation [2]. In atherosclerosis, blood flow through the vessels is reduced due to the thickening of the capillary basement membrane, loss of vascular elasticity, and deposition of lipids within the arterial walls [2]. This impaired vascular regulation leads to small vessel ischemia, further compromising tissue perfusion and slowing the recovery of any wounds the patient may sustain. Even minor injuries, such as superficial scratches, can demonstrate delayed healing due to inadequate blood supply.

Once a patient develops an ulcer, the prognosis can vary depending on glycemic control, adherence to healthy lifestyle modifications, and the quality of wound care provided [1]. A poor prognosis is often associated with inadequate blood supply, secondary infection, prolonged ulcer duration, and recurrence [1]. In the case presented, the patient has a history of previous ulcers and is now suffering from a mixed polymicrobial infected ulcer containing *Bacteroides fragilis*, *Streptococcus agalactiae* (group B *Streptococcus*), and *Staphylococcus aureus*.

*S. aureus* is part of the normal skin flora and is commonly identified when culturing the skin surface. Group B *Streptococcus* is typically found in the intestinal and lower genital tracts. *B. fragilis*, a member of the normal colonic microbiota, can spread beyond the gastrointestinal tract if the mucosal barrier is disrupted [3]. Once in the bloodstream, it may disseminate to adjacent tissues and cause clinically significant infections [3]. The presence of *B. fragilis* is often expected in long-standing, deep, or necrotic ulcers [4]. It is commonly associated with chronic polymicrobial infections, which may present with tissue ischemia, a foul odor, or gangrene [4].

When a patient presents with an ulcer, it is important to initiate empiric antibiotic therapy promptly to help prevent the progression and worsening of the infection. While cultures are being obtained to identify the causative microorganisms, broad-spectrum antibiotics are typically started to provide immediate coverage. It is not uncommon to choose trimethoprim-sulfamethoxazole (SXT) as empiric therapy because it offers fairly broad-spectrum coverage, is well absorbed via the oral route, and is relatively inexpensive [5]. Once the culture and sensitivity results are available, the antibiotic regimen can then be adjusted accordingly to target the specific organisms involved and address their resistance patterns. This stepwise approach ensures timely treatment while allowing for tailored therapy that optimizes patient outcomes.

## Case presentation

A 44-year-old man with a history of uncontrolled type 2 diabetes mellitus presented to the clinic on July 22, 2025, for a routine follow-up. He reported a pruritic lesion on the left lower leg that worsened following hot showers and scratching. The patient had been self-treating the lesion with triple antibiotic ointment (bacitracin, neomycin, and polymyxin B), along with topical iodine, but delayed seeking medical care. By the time of presentation, the lesion had progressed to a full-thickness ulcer. He recalled a similar episode on the right leg in 2023, which was resolved with clotrimazole cream. On examination, a shallow ulcer with well-demarcated edges, surrounded by rough skin and mild serous discharge, was observed on the medial aspect of the left lower leg. The surrounding tissue demonstrated decreased sensation and poor hair growth, findings consistent with a diabetic ulcer. A wound culture was obtained for microbiological analysis. The patient was advised on wound hygiene and pressure avoidance and referred to specialized wound care for ongoing management. Empiric antibiotic therapy with SXT was initiated.

Two weeks later, on August 8, 2025, the patient returned for a follow-up appointment to review lab and wound culture results. He reported that the lesion had improved, with resolution of the pruritus. He had been compliant with hydroxyzine and reported that he was no longer scratching the area. On physical examination, the ulcer was dry and healing. There was no erythema, warmth, or discharge noted. However, microbiology results from the wound culture revealed a polymicrobial infection with *B. fragilis*, group B *Streptococcus,* and *S. aureus*, resistant to SXT (Table 1, Table 2, and Table 3). 

**Table 1:** Anaerobic wound culture and Gram stain report from the left leg lesion demonstrating the heavy growth of Bacteroides fragilis. Gram stain shows moderate white blood cells, few epithelial cells, many Gram-positive cocci, and moderate Gram-negative bacilli, consistent with a mixed aerobic and anaerobic infection.

Culture, anaerobic bacteria with Gram stain	
Micro number	24780107
Test status	Final
Specimen source	Left leg lesion
Specimen quality	Adequate
Gram stain	Moderate white blood cells seen. Few epithelial cells. Many Gram-positive cocci. Moderate Gram-negative bacilli
Results	Heavy growth of *Bacteroides fragilis*

**Table 2:** Anaerobic culture of the left lower extremity lesion revealing the heavy growth of Staphylococcus aureus and group B Streptococcus.

Culture, anaerobic bacteria	
Micro number	2478018
Test status	Final
Specimen source	Left leg lesion
Specimen quality	Adequate
Results	Heavy growth of *Staphylococcus aureus*. Heavy growth of group B *Streptococcus* isolated

**Table 3:** Antibiotic susceptibility profile for Staphylococcus aureus isolated from the left leg lesion. Results show susceptibility to ciprofloxacin, clindamycin, erythromycin, gentamicin, moxifloxacin, oxacillin, tetracycline, and vancomycin, with resistance noted to trimethoprim-sulfamethoxazole. ^1 ^represents a laboratory reporting notation and does not affect the clinical interpretation. The isolate remains classified as susceptible, and no change in interpretation is implied. S: susceptible; R: resistant; INT: intermediate; MIC: minimum inhibitory concentration

Susceptibility testing	S. aureus	
	INT	MIC
Ciprofloxacin	S	≤0.5
Clindamycin	S	≤0.25
Erythromycin	S	≤0.25
Gentamicin	S	≤0.5
Moxifloxacin	S	≤0.25
Oxacillin	S	0.5^1^
Tetracycline	S	≤1
Trimethoprim-sulfamethoxazole	R	≥320
Vancomycin	S	≤0.5

Management involved the initiation of metronidazole and ciprofloxacin based on microbial sensitivity, along with twice-daily wound care using Hibiclens (chlorhexidine) and cold water while explicitly avoiding warm water to reduce irritation. The patient was advised to wear protective clothing at work to minimize the risk of trauma to the affected area. To mitigate potential gastrointestinal side effects of antibiotic therapy, probiotic yogurt was recommended. Additionally, high-dose metformin (administered three times daily) was prescribed to optimize glycemic control, alongside a high-intensity statin (atorvastatin 80 mg) for the management of hypercholesterolemia. The patient also received lifestyle counseling focused on dietary improvements and increased physical activity. A follow-up appointment was scheduled for two weeks later to assess progress and response to treatment.

At follow-up on August 29, 2025, the patient reported improvement in the ulcer and noted only pain localized to the area during prolonged standing. He remained compliant with medications and wound care. On physical examination, the ulcer was healing, with no signs of recurrent infection. The updated management plan included the discontinuation of antibiotic therapy to facilitate the recovery of the gastrointestinal flora. Wound hygiene was maintained with the continued use of chlorhexidine and air exposure during periods of rest, while protective bandage wrapping was advised during active hours to prevent further irritation or trauma. The patient was instructed to consume two servings of yogurt daily to support gut microbiome restoration. Given the patient's elevated A1c of 10.4%, dapagliflozin (Farxiga) was initiated to provide supplementary glycemic control.

## Discussion

Pathogens are frequently evolving, mutating, and gaining resistance, requiring the constant need to update medication usage. The favored treatment for skin and soft tissue infections (SSTIs) was originally β-lactams, because these medications targeted both group A *Streptococcus* and *S. aureus* as a single medication [5]. However, there has been a current shift to non-β-lactams, such as clindamycin, tetracyclines, and SXT, due to the global rise of methicillin-resistant *S. aureus* (MRSA) [5]. Now, healthcare professionals often choose non-β-lactams as the first choice for the empirical treatment of SSTIs, especially in cases where MRSA is common [5,6]. SXT is one popular choice. However, this case and recent research may suggest a need to reform the use of SXT as empiric or first-line therapy for MRSA-infected SSTIs due to increasing resistance patterns.

While resistance rates vary, several studies suggest SXT-resistant *S. aureus* is on the rise. The current system for national surveillance of drug resistance patterns lacks the assessment of *S. aureus* resistant to non-β-lactams [5]. Therefore, there is no national standard for the rate of SXT resistance, but several studies have explored this concept. A 2022 study analyzing SXT antibiotic susceptibility among three national inpatient databases found an increase in SXT-resistant MRSA from 3.5% in 2012 to 7.9% in 2017 [7]. Similarly, a cross-sectional study done in the Veterans Health Administration outpatient clinics saw SXT-resistant MRSA rising from 2.6% in 2010 to 9.2% in 2019 [8]. The antimicrobial resistance surveillance program, Assessing Worldwide Antimicrobial Resistance and Evaluation (AWARE), found *S. aureus* resistance to SXT to be roughly stable with 2.2% of isolates resistant in 2010 and 3.1% in 2016 [9]. Still, there was a slight increase. With increasing resistance rates and uncertainty among percentages, it is imperative that healthcare providers carefully consider their use of SXT.

It is also important for providers to maintain close patient follow-up and monitoring for antimicrobial resistance. In this case, we have a diabetic patient presenting with a skin infection of the lower leg. A recent meta-analysis reported that the most identified microorganism in diabetic lower limb infections was *S. aureus*, of which 18% was methicillin-resistant [9]. With our patient's high likelihood of a MRSA infection, he was prescribed SXT empirically. However, upon follow-up, it was found that the lesion had not fully healed and culture results/antimicrobial susceptibility testing identified SXT-resistant *S. aureus*. After adjustment of medications, the patient's infection improved. The initial failure of SXT in this case led to the further propagation of infection, which could have led to severe complications had management not been appropriately adjusted. This example of failed SXT therapy suggests that physicians must be diligent when treating infected diabetic ulcers and may no longer be able to rely on trusted therapies.

Tailoring antibiotic coverage based on culture and sensitivity results is one such way to avoid antimicrobial resistance. Further ways to combat increasing MRSA antibiotic resistance include the use of current antibiograms, selecting appropriate antibiotic regimens based on local resistance patterns, and monitoring patients for treatment failure [5]. Treating SSTIs in accordance with these methods can prevent worsening infections caused by antibiotic resistance and improve patient outcomes.

## Conclusions

This case of a 44-year-old man with poorly controlled type 2 diabetes mellitus and a polymicrobial diabetic foot ulcer demonstrates the flaws of utilizing empiric SXT as first-line therapy for diabetic foot infections and how early culture-guided therapy can have a huge impact on patient outcomes. Cultures showed *S. aureus* resistant to SXT, *B. fragilis,* and group B *Streptococcus*, leading to a change in treatment plan to metronidazole and ciprofloxacin, more frequent wound care with chlorhexidine, as well as optimizing the patients' metformin and statin doses. Following this pivot in treatment at the two-week follow-up, the patient showed clinical improvement and resolution of the infection, and the antibiotics were discontinued. Although severe adverse outcomes were avoided in this case, SSTIs remain a main contributor to morbidity in diabetic patients. These infections have serious complications and effects on quality of life, often leading to chronic wounds and limb amputations. Due to the severity of SSTIs in the diabetic population, ensuring successful treatment is of the utmost importance.

Generally, diabetic foot infections are often sub-optimally managed when the local microbial and resistance patterns are not considered. Increasing SXT resistance among MRSA warrants the careful selection of empiric antibiotics, being diligent with follow-ups to detect early treatment failure, and timely adjustment based on culture and sensitivity results. Providers should align their regimens with current antibiograms and report notable trends whether it be of resistance or notable pathogens.
